# Effect of Ethanol Consumption on the Placenta and Liver of Partially IGF-1-Deficient Mice: The Role of Metabolism via CYP2E1 and the Antioxidant Enzyme System

**DOI:** 10.3390/biology11091264

**Published:** 2022-08-25

**Authors:** Irene Martín-Estal, Óscar R. Fajardo-Ramírez, Mario Bermúdez de León, Carolina Zertuche-Mery, Rodolfo Benavides-Guajardo, María Isabel García-Cruz, Julieta Rodríguez De Ita, Inma Castilla-Cortázar, Fabiola Castorena-Torres

**Affiliations:** 1 Tecnologico de Monterrey, Escuela de Medicina y Ciencias de la Salud, Ave. Morones Prieto 3000, Monterrey 64710, N.L., Mexico; 2 Departamento de Biología Molecular, Centro de Investigación Biomédica del Noreste, Instituto Mexicano del Seguro Social, Monterrey 64720, N.L., Mexico; 3 Fundación de Investigación HM Hospitales, 28015 Madrid, Spain

**Keywords:** IGF-1 deficiency, ethanol, placenta, antioxidant enzyme system, CYP2E1

## Abstract

**Simple Summary:**

Ethanol is the most consumed drug worldwide, even during pregnancy. One of its adverse outcomes is fetal growth restriction, an alteration in development due to decreased IGF-1 levels. Several studies have shown that ethanol can impair the IGF-1 signaling pathway, thus exacerbating IGF-1 adverse effects in both intrauterine and postnatal growth and development. In this manuscript, we used a partially IGF-1-deficient mouse model to demonstrate the key role of IGF-1 in fetal development, as well as ethanol’s adverse effects on CYP2E1 expression levels and the antioxidant enzyme system during pregnancy.

**Abstract:**

Ethanol use during pregnancy is a risk factor for developing adverse outcomes. Its metabolism by cytochrome P450 2E1 (CYP2E1) produces radical oxygen species (ROS), promoting cellular injury and apoptosis. To date, no studies have been conducted to elucidate the teratogenic effects due to both IGF-1 deficiency and ethanol consumption in mice placentas. The aim of this study is to determine the effect of ethanol consumption on the placenta and liver of partially IGF-1-deficient mice, the role of metabolism via CYP2E1, and the antioxidant enzyme system. Heterozygous (HZ, *Igf1^+/−^*) pregnant female mice were given water or 10% ethanol. Wild-type (WT, *Igf1^+/+^*) female mice were used as controls. At gestational day 19, pregnant dams were euthanized, and maternal liver and placentas were collected. Pregnant HZ dams were smaller than controls, and this effect was higher due to ethanol consumption. *Cyp2e1* gene was overexpressed in the liver of HZ pregnant dams exposed to ethanol; at the protein level, CYP2E1 is reduced in placentas from all genotypes. The antioxidant enzymatic system was altered by ethanol consumption in both the maternal liver and placenta. The results in this work hint that IGF-1 is involved in intrauterine development because its deficiency exacerbates ethanol’s effects on both metabolism and the placenta.

## 1. Introduction

The placenta is a vital organ for both mother and fetus, as it allows the exchange of nutrients and waste products between them, and secretes several hormones, especially the cytotrophoblast and the syncytiotrophoblast layers within the junctional zone. These hormones are involved in the establishment and maintenance of pregnancy and fetal development, such as insulin-like growth factor 1 (IGF-1) and insulin-like growth factor 2 (IGF-2) [1]. For this reason, both hormones play a crucial role during gestation; and an abnormal secretion is associated with fetal growth restriction (FGR) [2]. Recently, FGR has been described as an IGF-1 deficiency condition, as shown in numerous human studies, where *Igf1* gene mutations lead to an altered IGF-1 downstream signaling, resulting in postnatal growth failure and developmental delay, sometimes accompanied by sensorineural deafness and intellectual deficit [3].

There are numerous substances that can damage the functionality and morphology of the placenta, e.g., ethanol. To date, it continues to be the most consumed drug worldwide, even among pregnant women, despite its well-known adverse outcomes during pregnancy, such as fetal death, miscarriage, FGR, low birth weight, premature birth, and Fetal Alcohol Spectrum Disorders (FASD) [4].

Ethanol is metabolized in the liver to acetaldehyde via oxidative pathways: 90% approximately by ethanol dehydrogenase (ADH) and 10% by cytochrome P450 2E1 (CYP2E1), an isoform of the cytochrome P450 system (CYP) [5]; then, acetaldehyde is converted to acetate by aldehyde dehydrogenase (ALDH) and enters the Krebs cycle to produce water and carbon dioxide (CO_2_). Additionally, catalase (CAT) can metabolize ethanol in the presence of a hydrogen peroxide (H_2_O_2_)-generating system, such as the enzyme complex NADPH oxidase or the enzyme xanthine oxidase [6]. As a result of ethanol metabolism by CYP2E1, localized in the junctional zone of the placenta [7], reactive oxygen species (ROS) are produced, triggering DNA damage and promoting the oxidation of lipids, proteins, and other metabolites [8]. This increase in ROS production can compromise the activity of antioxidant enzymes such as superoxide dismutase (SOD), CAT, and glutathione peroxidase (GPx), activating the intrinsic apoptotic pathway and promoting mitochondrial damage [9].

At present, several studies in animal models of ethanol consumption suggest that ethanol alters the IGF-1 signaling pathway since it decreases fetal weight [10] and reduces IGF-1 and insulin-like growth factor binding protein 3 (IGFBP-3) plasmatic concentrations in both mother and fetus [11]. In addition, insulin and IGF-1 receptor tyrosine kinase activities are also decreased in these models [12]; and gene expression and/or secretion of insulin, IGF-1, and IGF-2 are impeded [10]; impairing downstream IGF-1 signaling transduction.

The present study aims to determine the effects of ethanol consumption in the liver and placenta from partially IGF-1-deficient mice on CYP2E1 expression levels and antioxidant enzyme system.

## 2. Materials and Methods

### 2.1. Animals and Experimental Design

Over the years, our group has developed a mouse model with partial IGF-1 deficiency (HZ) that displays weight reduction and lower serum levels of IGF-1 than controls, among other pathologies [13]. Briefly, IGF-1 heterozygous mice were obtained by crossbreeding transgenic mice, line 129SV and Igf1^tm1Ts^/ImJ (003258, Jackson Laboratory, Maine, USA) and CD1 (non-consanguineous, Circulo A.D.N S. A de C.V., Mexico City, Mexico). A neomycin selection cassette was inserted into exon 3 after codon 15. This mutation inserts multiple stop codons into all reading frames of the B chain of the encoded protein.

The conditions of a 12 h light/dark cycle, constant humidity (50–55%), and temperature (20–22 °C) were implemented to house the animals. Both food (PicoLab^®^ Rodent Diet 20 5053 *, MO, USA) and water were given ad libitum. All of the experimental procedures were performed in compliance with the National Institutes of Health guide for the care and use of laboratory animals (NIH Publications No. 8023, revised 1978) and the Norma Oficial Mexicana (NOM-062-ZOO-1999) for technical specifications to produce, care, and use laboratory animals. Furthermore, all of the experimental procedures were approved by the Institutional Committee for the Care and Use of Laboratory Animals of Tecnologico de Monterrey (Protocol 2018-006).

**Figure 1:**
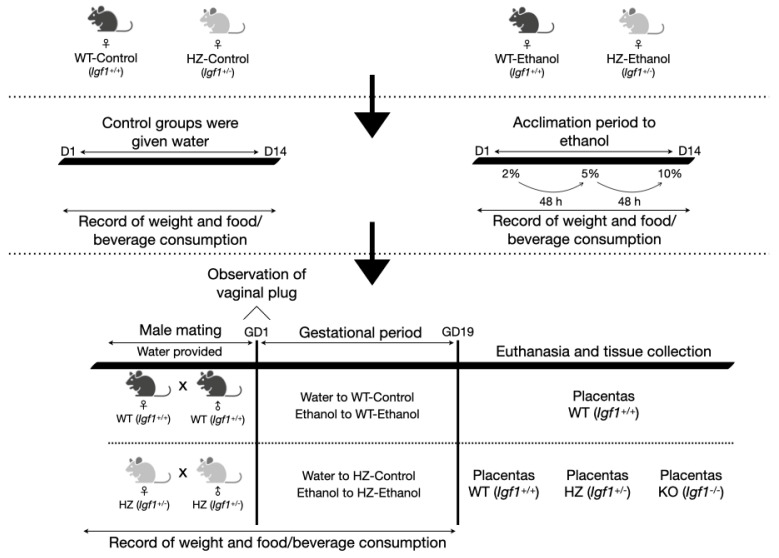
Diagram of the experimental procedure. D: day; GD: gestational day; HZ: heterozygous; KO: knock-out; WT: wild-type.

Wild-type (WT, *Igf1^+/+^*) and heterozygous (HZ, *Igf1^+/−^*) female mice 16 ± 8 weeks old were divided into four experimental groups: two control groups given water (WT-Control, *n =* 6; and HZ-Control, *n =* 6), and two other groups treated with food grade ethanol (WT-Ethanol, *n = 4*; and HZ-Ethanol, *n* = 9) (purity 96%, LaFe, Nuevo Leon, Mexico) (Figure 1).

Ethanol exposure was performed according to the protocol described by Kleiber et al. [14]. Firstly, the female mice (WT and HZ) were exposed for 14 days to increasing concentrations of 2%, 5%, and 10% ethanol in drinking water, each introduced after a 48-h acclimation to the previous concentration (Figure 1). During this period, the control WT and HZ female mice were given water.

Following the 14-day ethanol acclimation period, the WT and HZ female mice from all experimental groups were mated overnight with WT and HZ male mice 16 ± 8 weeks old, respectively (Figure 1). During mating, only water was provided in all experimental groups to prevent the males from consuming ethanol. At the end of the mating period, the males were removed, and the females were examined for the presence of the vaginal plug, indicating gestational day 1. Nonpregnant females from the water-exposed groups (WT-Control and HZ-Control) were mated again until the presence of the vaginal plug was observed. Conversely, the nonpregnant females from the ethanol-exposed groups (WT-Ethanol and HZ-Ethanol) were given 10% ethanol for 24 h and then mated once again with the male mice. If the vaginal plug was not observed after this second mating, the ethanol-exposed female mice were euthanized by cervical dislocation.

During gestation, the pregnant females were given 10% ethanol (WT-Ethanol and HZ-Ethanol) or water (WT-Control and HZ-Control) until day 19 post coitum. All through the 14-day ethanol acclimation period and gestation, the female mice were weighed to record the weight gain before and after gestation with both treatments (water and ethanol), food and beverages were also monitored to observe their consumption throughout both periods. On day 19 post coitum, the pregnant females were euthanized by cervical dislocation (Figure 1). Following, a blood sample was obtained by cardiac puncture. Afterwards, livers, placentas, and fetuses (from HZ × HZ matings) were recovered, measured, and weighed from pregnant dams after caesarean section. The tails from all of the fetuses were cut for sexing and genotype determination. Following, the placentas and mothers’ livers were stored in liquid nitrogen for immunoblot, reverse transcription coupled to quantitative polymerase chain reaction (RT-qPCR), and antioxidant status determinations; or in 4% paraformaldehyde for conventional histology and immunohistochemical studies.

### 2.2. Sexing and Genotyping of Animals

To sex and genotype the animals by conventional polymerase chain reaction (PCR) (Veriti 96-well Thermal Cycler, Applied Biosystems, Foster City, CA, USA), DNA extraction was performed from tails of fetuses, stored at −20 °C, using the Wizard Genomic DNA Purification Kit (A1125, Madison, Promega, WI, USA) following the manufacturer’s instructions.

Specific primers were used to identify the *Igf1* gene (WT Forward: 5′-TTC ATG CCA CAC TGC TCT TC-3′; Common: 5′-AGA GGG GAT GGG AGA GCT AC-3′; and Mutant Forward: 5′-GCC AGA GGC CAC TTG TGT AG-3′), for animal genotyping; and sex-determining region protein gene (*Sry*) (Forward: 5′-GAG TAC AGG TGT GCA GCT CT-3′; Reverse: 5′-TGG GAC TGG TGA CAA TTG TC-3′) for animal sexing; and beta actin (*Actb*) gene (Forward: 5′-AGC CAT GTA CGT AGC CAT CC-3′; Reverse: 5′-TGT GGT GGT GAA GCT GTA GGC-3′) as an endogenous control. All of the primers were acquired from IDT (Iowa, USA). Conventional PCR analysis was performed using the GoTaq Green Master Mix (M712C, Promega, WI, USA) following the manufacturer’s instructions.

### 2.3. Gene Expression Studies via RT-qPCR

The placental and liver tissues from all of the experimental groups were cryopreserved in liquid nitrogen, as previously mentioned. On the day of the PCR determinations, the placental and liver samples were homogenized with Trizol reagent (15596026, Invitrogen, Waltham, CA, USA), and the total RNA was extracted. The RNA purity was verified using the A260/A280 ratio (Nanodrop 2000 Spectrophotometer, Thermo Scientific, Waltham, Massachusetts, USA), and the ribosomal subunits were visualized in a 1% agarose gel stained with Gel red (410035x, Biotium, CA, USA). The isolated RNA was stored at −80 °C until analysis. Then, the complementary DNA (cDNA) was prepared from 500 ng of the total RNA using the SuperScript III First-Strand Synthesis System (Invitrogen, CA, USA) according to the manufacturer’s instructions.

TaqMan Universal PCR Master Mix (Thermofisher, Waltham, MA, USA) was used for the PCR analyses in a total volume of 20 µL containing 400 nM of each oligonucleotide and 200 mM of specific Taqman^®^ probes for the *Cyp2e1* gene (Mm00491122_m1, supplied by Applied Biosystems, CA, USA). The assays were performed in a 96-well reaction plate on a Quant Studio 3.0 thermocycler (Applied Biosystems, Foster City, CA, USA). Ribosomal 18S RNA amplification was used as an endogenous control (Applied Biosystems, Foster City, CA, USA).

Each sample was run in triplicate, and the negative controls were included in the same plate. The results were analyzed using the comparative threshold cycle method for relative gene expression [15].

### 2.4. Immunoblotting Analysis

Immunoblotting analysis was used to examine CYP2E1 and 4-hydroxynonenal (4-HNE) protein expression levels in the liver from pregnant dams.

Protein was extracted from the liver tissue homogenates using Hepes lysis buffer in a sterile mortar containing protease-inhibitor cocktail set III and phosphatase-inhibitor cocktail set I (Calbiochem, EMD Biosciences, CA, USA). Subsequently, the protein samples were mixed in Laemmli buffer (161-0747, Bio-Rad, CA, USA), boiled for 7 min, and then equal amounts (100 μg per lane) were fractioned on a polyacrylamide gel and transferred onto a polyvinylidene fluoride (PVDF) membrane (BioRad, Hercules, CA, USA) using a Trans-Blot^®^ SD Semi-Dry Transfer Cell apparatus (BioRad, Hercules, CA, USA).

Membrane blocking was carried out for 1 h in 5% non-fat dry milk diluted in 10 mM phosphate-buffered saline (PBS). Then, primary antibodies were added to the samples and diluted in 10 mM PBS buffer containing 0.1% Tween 20 and 5% bovine serum albumin (BSA): rabbit anti-CYP2E1 (ab28146, Abcam, Cambridgeshire, UK) diluted 1:1000; rabbit anti-4-HNE (ab46545, Abcam, Cambridgeshire, UK) diluted 1:1000; or rabbit anti-beta Actin (ab8227, Abcam, Cambridgeshire, UK) diluted 1:1000, the latter being an endogenous control. The samples were incubated overnight at 4 °C with the abovementioned primary antibodies. After three washes in 10 mM PBS containing 0.1% Tween 20, secondary Horseradish Peroxidase (HRP) conjugate antibody (goat Anti-Rabbit IgG H&L, ab205718, Abcam, Cambridgeshire, UK) diluted 1:10,000 was added, followed by three washes with 10 mM PBS containing 0.1% Tween 20.

The blots were detected with ECL™ Blotting Reagents (GERPN2109, Sigma-Aldrich, MO, USA) and developed using the ChemiDoc XRS+ System (BioRad, Hercules, CA, USA).

To incubate the same blot with different primary antibodies, after the detection of the blots with ECL™ Blotting Reagents, the stripping of the membranes was realized. Briefly, the membranes were incubated in a stripping buffer (15 g of glycine, 1 g of sodium dodecyl sulfate (SDS), and 10 mL of Tween 20, pH 2.2) for 10 min at room temperature and then washed twice in 10 mM PBS and in Tris-buffered saline and 0.1% Tween 20. Lastly, the membranes were incubated with another primary antibody and the regular process of immunoblotting was conducted as aforementioned.

### 2.5. Immunohistochemical Analysis

The sections (4 μm) of the paraffin-embedded placental tissues were deparaffinized and rehydrated in decreasing ethanol concentrations. The sample sections were incubated with 3% hydrogen peroxide in darkness at room temperature for 30 min to inactivate endogenous peroxidases. The retrieval of the antigen was induced with 2 mM ethylenediaminetetraacetic acid (EDTA) pH 8.0 and 50 mM Tris-HCl pH 9.0 by microwave heating at 100 °C for 8 min. The sections were incubated overnight at 4 °C with specific primary antibodies: rabbit anti-CYP2E1 (ab28146, Abcam, Cambridgeshire, UK) diluted 1:1000; or rabbit anti-beta Actin (ab8227, Abcam, Cambridgeshire, UK) diluted 1:1000, the latter being an endogenous control. Then, the sections were incubated with the secondary HRP conjugate antibody (goat Anti-Rabbit IgG H&L, ab205718, Abcam, Cambridgeshire, UK) diluted at 1:5000 for 1 h at room temperature to detect the primary antibodies. Staining was developed using Steady diaminobenzidine (DAB)/Plus kit (ab103723, Abcam, Cambridgeshire, UK) following the manufacturer’s instructions. The negative controls were conducted by omitting the primary antibody.

Digital images of tissue sections were captured by three observers (double-blind) using a Zeiss Axio Imager M2 microscope.

### 2.6. Antioxidant Enzymatic Status

CAT, GPx, and SOD were measured in the placental and liver tissue homogenates using Cayman Assay Kits for each enzyme (707002, 703102, and 706002, Cayman Chemical, Ann Arbor, MI, USA, respectively), following the manufacturer’s procedure.

### 2.7. Statistical Analysis

All of the data are represented by mean ± standard deviation (SD). Significance was estimated with non-parametric (Mann–Whitney U and Kruskal–Wallis tests) or parametric (Student *t*-test and ANOVA) statistical tests, using Mann–Whitney U test or Student *t*-tests to compare the two groups, whereas comparisons among more than two groups were carried out using the Kruskal–Wallis test or ANOVA. To evaluate the interaction of the two independent variables in the present study (ethanol consumption and IGF-1 deficiency), a general linear model was performed. Differences were considered significant at a level of *p <* 0.05 (significance levels for multiple testing were adjusted). Statistical analyses were performed on SPSS 26 (IBM, NY, USA), and graphs were generated on Prism 8.2.1 (GraphPad Software, San Diego, CA, USA).

## 3. Results

### 3.1. The Effect of Ethanol Consumption in Maternal Parameters before and after Gestation

The maternal parameters, such as weight gain and average food and beverage consumption, were analyzed during the 14-day ethanol acclimation and gestational periods. No significant differences were found in these parameters throughout the 14-day ethanol acclimation period in all experimental groups when compared to the WT-Control group (Supplementary Table S1). On gestational day 1, pregnant dams from the HZ-Control group had a lower body weight than the WT-Control (*p* < 0.05) and the WT-Ethanol dams *(p* < 0.01); similarly, the pregnant HZ dams exposed to ethanol (HZ-Ethanol) had lower body weights than the WT-Ethanol dams (*p* < 0.05) (Supplementary Table S2).

**Figure 2:**
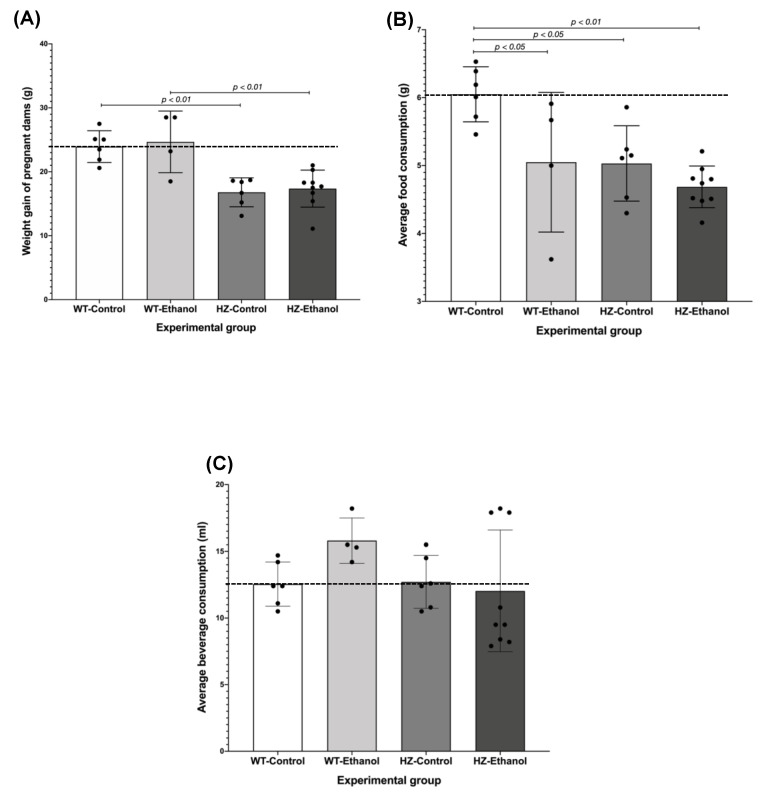
Effect of gestational ethanol consumption in maternal parameters: (**A**) weight gain, (**B**) average food consumption, and (**C**) average beverage consumption. Samples sizes were: WT-Control, *n* = 6; WT-Ethanol, *n* = 4; HZ-Control, *n* = 6; HZ-Ethanol, *n* = 9. Black dots represent the data dispersion. The statistical tests used were Student *t*-test and U Mann–Whitney test to compare wild-type (WT) and heterozygous (HZ) mice, and ANOVA or Kruskal–Wallis test to compare all experimental groups. Differences were considered significant when *p* < 0.05.

On gestational day 19, the weight gain of the pregnant dams during gestation was compared between all groups, and it was found that both HZ groups (HZ-Control, 16.78 g ± 0.92; and HZ-Ethanol, 17.37 g ± 0.96) weighed less than the WT groups (WT-Control, 23.94 g ± 1.01; and WT-Ethanol, 24.67 g ± 2.41; *p* < 0.001), and this effect was not altered by the ethanol treatment (Figure 2A), suggesting that partial IGF-1 deficiency is responsible for the lower weight gain in pregnant females throughout pregnancy. Interestingly, partial IGF-1 deficiency reduced the average food consumption in the control group (HZ-Control, 5.03 g ± 0.23; *p* < 0.05) compared to the WT-Control group (Figure 2B). Additionally, the average food consumption was significantly reduced due to ethanol consumption in the WT dams (WT-Ethanol, 5.05 g ± 0.51) compared to the WT-Control group (6.05 g ± 0.17; *p* < 0.05) (Figure 2B). Moreover, the synergic effect of both partial IGF-1 deficiency and ethanol consumption (HZ-Ethanol, 4.69 g ± 0.10) during gestation decreased the average food consumption compared to the WT-Control group (*p* < 0.01). These results in average food consumption correspond to those obtained in the ratio between food consumption and body weight (Table 1). No significant differences were found in beverage consumption throughout gestation in all experimental groups (Figure 2C).

On gestational day 19, prior to the euthanasia of the pregnant dams, maternal body weight was recorded, and no significant differences were observed due to ethanol consumption between both WT and HZ groups; whereas the presence of partial IGF-1 deficiency (HZ-Control, 40.73 g ± 1.28) showed a reduction in maternal body weight compared to the WT-Control (52.05 g ± 1.21; *p* < 0.05) and the WT-Ethanol groups (51.73 g ± 5.82; *p* < 0.05) (Table 1). Ethanol consumption did not affect liver weight in pregnant dams from all experimental groups (Table 1). The liver-to-body-weight ratio was higher in the ethanol-treated groups (WT-Ethanol and HZ-Ethanol, *p* < 0.05) than in the control groups (WT-Control and HZ-Control) (Table 1). Remarkably, a positive and significant correlation was found between maternal body and liver weights in the HZ-Ethanol group (Pearson’s r = 0.919; *p* < 0.001).

### 3.2. The Effect of Ethanol Consumption in Fetal and Placental Parameters

To evaluate the effect of IGF-1 deficiency and chronic ethanol exposure, the WT, HZ, and knockout (KO) fetuses and placentas obtained from HZ x HZ matings were analyzed (Figure 1). No significant differences were observed in fetal and placental parameters due to the ethanol exposure of dams during gestation. To evaluate the effect of both IGF-1 deficiency and ethanol consumption on the fetal parameters, a general linear model was performed. In this sense, the presence of KO genotype and ethanol consumption affected fetal height (*r*^2^ = 0.504) and weight (*r*^2^ = 0.592), *p* < 0.001 in both cases, when compared to the WT and HZ genotypes (Table 2). Furthermore, ethanol consumption altered fetal height in WT genotype compared to the WT-Control (*r*^2^ = 0.504, *p* < 0.05) and fetal weight in the HZ genotype compared to the HZ-Control (*r*^2^ = 0.592, *p* < 0.05) (Table 2). Interestingly, the presence of the KO genotype and ethanol consumption significantly increased placental weight compared to the KO-Control (*r*^2^ = 0.070, *p* < 0.05) (Table 2).

### 3.3. The Effect of Ethanol Consumption in Placental and Hepatic Expression of CYP2E1

**Figure 3:**
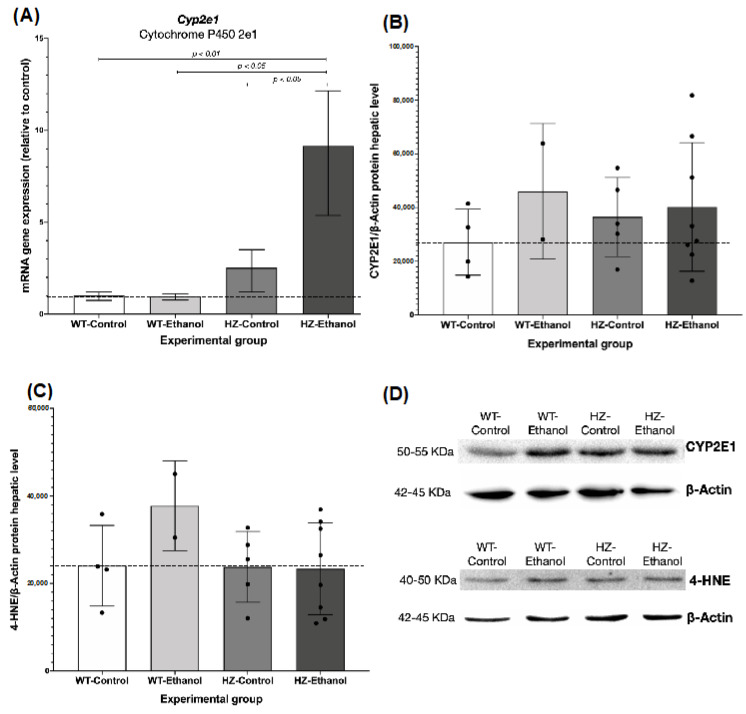
Effect of ethanol consumption during gestation in: (**A**) Cytochrome P450 2E1 (*Cyp2e1*) gene expression by reverse transcription coupled to polymerase chain reaction (RT-qPCR) in liver from pregnant dams from all experimental groups (WT-Control, *n* = 3; WT-Ethanol, *n* = 2; HZ-Control, *n* = 4; HZ-Ethanol, *n* = 5); (**B**) CYP2E1 and (**C**) 4-hydroxynonenal (4-HNE) protein expression levels by immunoblot in liver from pregnant dams from all experimental groups (WT-Control, *n* = 4; WT-Ethanol, *n* = 2; HZ-Control, *n* = 5; HZ-Ethanol, *n* = 8). Black dots represent data dispersion. The statistical tests used were Student *t*-test to compare wild-type (WT) and heterozygous (HZ) mice and ANOVA to compare all experimental groups. Differences were considered significant when *p* < 0.05. (**D**) Immunoblot representative images of CYP2E1 and 4-HNE protein levels in liver from pregnant dams from all experimental groups.

*Cyp2e1* gene expression was determined by RT-qPCR in the liver and placental tissues from the pregnant dams. Partial IGF-1 deficiency (HZ-Control and HZ-Ethanol) significantly increased *Cyp2e1* liver expression compared to the WT groups (WT-Ethanol and WT-Control; *p* < 0.05). In addition, an inducer effect was observed in the HZ-Ethanol dams in *Cyp2e1* liver expression when compared to the HZ-Control group (1.52-fold, *p* < 0.05) (Figure 3A). Interestingly, a synergic effect was observed in partial IGF-1-deficient mice chronically exposed to ethanol during gestation (HZ-Ethanol, 8.15-fold) compared with both the WT-Control (*p* < 0.01) and the WT-Ethanol groups (*p* < 0.05) (Figure 3A). In placentas, *Cyp2e1* expression levels were only detected in the WT-Control fetuses (data not shown).

Regarding the protein levels in the liver, when grouped by treatment, a significant difference in CYP2E1 expression was found between the ethanol (WT-Ethanol and HZ-Ethanol) and control (WT-Control and HZ-Control) groups (*p* < 0.05), but no significant differences were observed when all of the experimental groups are compared to each other (Figure 3B,D, Supplementary Table S3 and Supplementary Figure S1).

**Figure 4:**
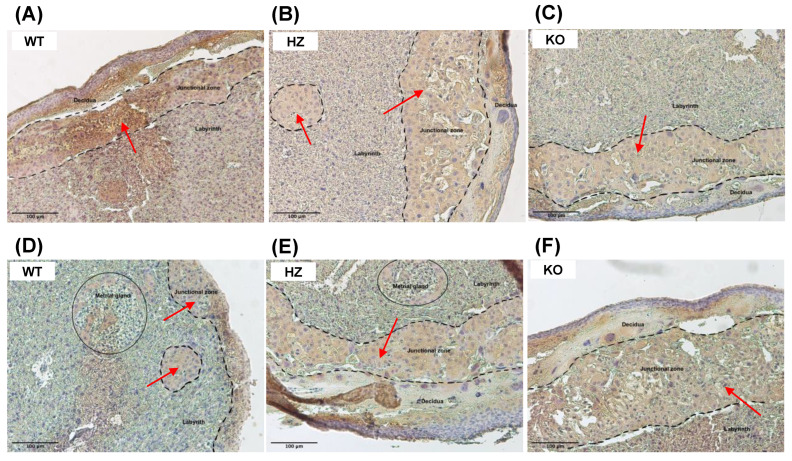
Effect of ethanol consumption during gestation in Cytochrome P450 2E1 (CYP2E1) levels analyzed by immunohistochemistry in placentas from wild-type (WT), heterozygous (HZ) and knock-out (KO) mice given water ((**A**), *n* = 5; (**B**), *n* = 6 and (**C**), *n* = 6) or ethanol ((**D**), *n* = 7; (**E**), *n* = 6 and (**F**), *n* = 5). Original magnification 100×. Scale bar = 100 μm. Representative images from one mouse of each group are included, where three principal layers of the placenta are observed: decidua, junctional zone, and labyrinth. Red arrows denote CYP2E1 expression in the junctional zone of the placenta, where trophoblast cells are expressed.

The immunohistochemistry analysis of CYP2E1 expression in the placentas showed a decrease in CYP2E1 placental expression with IGF-1 deficiency, in addition to remarkable structural changes in the junctional zone where trophoblast cells are expressed (Figure 4A–C). Likewise, ethanol consumption during gestation reduces CYP2E1 expression in placentas from all genotypes, exacerbating the structural changes (Figure 4D–F).

### 3.4. The Effect of Ethanol Consumption on the Oxidative Environment in Liver from Pregnant Dams

To explore the oxidative damage produced by either IGF-1 deficiency and/or ethanol consumption, 4-HNE was evaluated as a marker for lipid peroxidation. In this sense, 4-HNE levels showed no significant differences in all of the experimental groups (Figure 3C,D, Supplementary Table S4 and Supplementary Figure S2).

Interestingly, a positive and significant correlation was found between the CYP2E1 and 4-HNE levels in the livers of the WT-Ethanol pregnant dams (*Pearson’s r* = 1.000, *p* = 0.000).

### 3.5. Antioxidant Enzyme Activities

**Figure 5:**
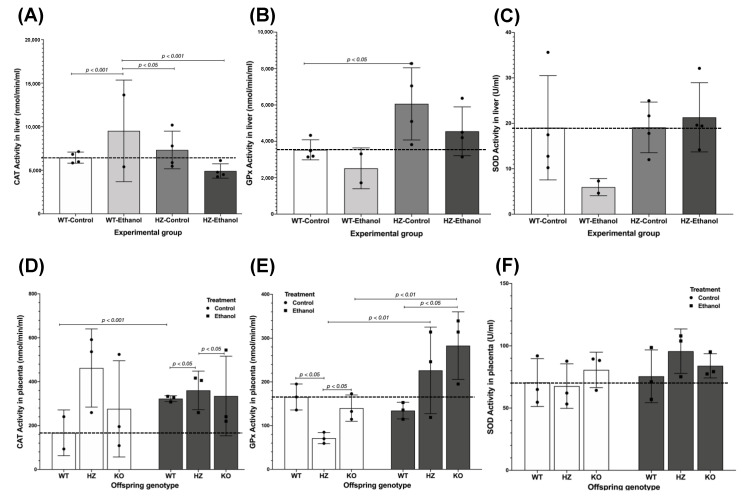
Effect of ethanol consumption throughout gestation in the antioxidant activities of catalase (CAT), glutathione peroxidase (GPx), and superoxide dismutase (SOD) in liver from pregnant dams (**A**–**C**), respectively) and placental tissues (**D**–**F**), respectively) from all experimental groups. Sample sizes for maternal livers were: WT-Control, *n* = 4; WT-Ethanol, *n* = 2; HZ-Control, *n* = 4; HZ-Ethanol, *n* = 4. Sample sizes for placentas from control and ethanol groups were WT, *n* = 3; HZ, *n* =3; KO, *n =* 3; for each treatment. Black dots represent the data dispersion. The statistical tests used were Student *t*-test to compare between two groups, ANOVA to compare between more than two groups, and general linear model to evaluate the effect of the two independent variables in the present study (ethanol consumption and IGF-1 deficiency). Differences were considered significant when *p* < 0.05.

The analysis of the antioxidant enzyme activities (CAT, GPx, and SOD) in the maternal liver among all of the experimental groups showed an interesting pattern related to ethanol exposure and not partial IGF-1 deficiency (Figure 5A–C). Ethanol consumption during gestation increased CAT activity in the livers of the WT pregnant dams (WT-Ethanol) compared to the WT-Control group (*p* < 0.001). The only significant differences in CAT activity were found in the HZ-Ethanol (*p* < 0.001) and the HZ-Control (*p* < 0.05) groups when compared to the WT-Ethanol group (Figure 5A). Regarding GPx activity, partial IGF-1 deficiency increased GPx activity in the livers of pregnant dams (HZ-Control, *p* < 0.05) compared to the WT-Control group (Figure 5B). The SOD activity levels did not show differences between all of the experimental groups, but ethanol seemed to decrease SOD levels in the WT group (WT-Ethanol) (Figure 5C).

The placental tissues showed an increase in CAT activity levels due to ethanol consumption (WT-Ethanol and HZ-Ethanol) compared to those groups given water (WT-Control and HZ-Control) (*p* < 0.05) (Figure 5D). To evaluate the effect of both IGF-1 deficiency and ethanol consumption on the placental antioxidant enzymatic system, a general linear model was performed. In this sense, ethanol consumption altered the CAT activity in the WT placentas compared to the WT-Control placentas (*r*^2^ = 0.008, *p* < 0.001) (Figure 5D). Moreover, the presence of IGF-1 deficiency and ethanol consumption affected CAT activity in the HZ and KO placentas compared to the WT ethanol-exposed placentas (*r*^2^ = 0.008, *p* < 0.05) and the HZ ethanol-exposed placentas (*r*^2^ = 0.008, *p* < 0.05), respectively (Figure 5D).

Similarly, an increase in GPx activity was found in placentas exposed to ethanol in all offspring genotypes (*p* < 0.05) (Figure 5E). When divided by genotype, significant differences were observed in the HZ and KO control placentas compared to the WT-Control placentas (*r*^2^ = 0.572, *p* < 0.05) (Figure 5E); also, in both HZ and KO placentas GPx activity was increased in the ethanol-exposed group compared to control groups given water (*r*^2^ = 0.572, *p* < 0.01), but no differences were observed in the WT placentas (Figure 5E). Interestingly, the KO ethanol-exposed placentas showed an increased effect in GPx activity compared to the WT ethanol-exposed placentas (*r*^2^ = 0.572, *p* < 0.05) (Figure 5E). No significant differences were found in SOD activity in the placentas from all of the experimental groups (Figure 5F).

## 4. Discussion

Over the years, our group has developed a mouse model with partial IGF-1 deficiency (HZ) that displays weight reduction and lower serum levels of IGF-1 than the controls, among other pathologies [13]. The results herein reported that the HZ pregnant dams had a lower body weight than the WT control mice on gestational day 19, hinting that the slight IGF-1 deficiency reduced both maternal and fetal weights, and this may aggravate the effects of ethanol on postnatal growth due to a greater vulnerability. In addition, ethanol consumption throughout gestation did not change these parameters.

**Figure 6:**
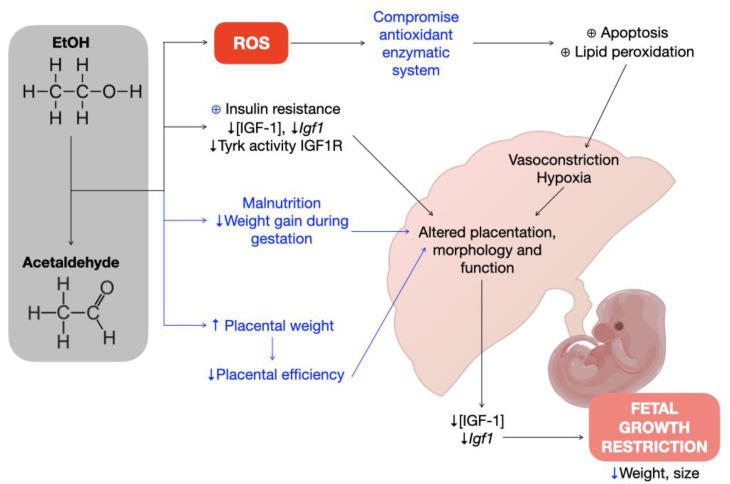
Hypothetical ethanol’s effect on placentation and fetal development and its association with insulin-like growth factor 1 (IGF-1). Blue lines represent the results obtained in the present project.

Ethanol consumption may increase the risk of micronutrient deficiencies, e.g., choline, folate, B12, and iron, particularly during pregnancy, leading to a greater fetal vulnerability to ethanol exposure, as shown in several experimental and clinical studies [16]. Moreover, differences in body composition, such as lower pre-pregnancy weights, exacerbated the effects of ethanol on postnatal growth [17]. This undernutrition in micronutrients could result in growth hormone (GH) resistance, as is shown in several experimental models, where a caloric restriction is associated with reductions in GH receptor mRNA transcription and, hence, insulin and IGF-1 serum concentrations [18]. The results herein have shown that the IGF-1 partially deficient pregnant dams (HZ) did not gain weight as much as WT dams during gestation, independently of ethanol consumption, having a reduced food consumption during this period, suggesting that this lower pre-pregnancy weight could promote fetal vulnerability to ethanol exposure (Figure 6). However, more studies are needed to confirm this hypothesis, as well as to understand the underlying mechanisms of IGF-1 deficiency, ethanol consumption, and micronutrient deficiencies throughout pregnancy.

Recently, IGF-1 deficiency has been associated with FGR, as shown in clinical and experimental models where low IGF-1 levels were found in both mother and fetus, which correspond with altered placental morphology and function [19]. The results in the present study showed that ethanol consumption throughout gestation decreases height and weight, especially in KO fetuses. On the other hand, it was observed that ethanol increases placental weight in KO fetuses compared to those KO given water, which aligns with other reports [20], suggesting that this effect could be due to congestion in the labyrinthine region of the placenta that may alter nutrient exchange surface, thus modifying placental efficiency and resulting in poor fetal nutrition and development [21] (Figure 6). However, more studies are needed to determine the teratogenic effects of ethanol consumption in the placenta.

Ethanol, due to its chemical structure, can diffuse across biological membranes, such as the placenta, where it accumulates in the amniotic fluid, thus exposing the fetus to the same ethanol concentration levels observed in the mother. CYP2E1, an isoform of the CYP450 family, is a monooxigenase that catalyzes the conversion of substrates, e.g., ethanol, into more polar metabolites, producing ROS as a consequence of such chemical reaction [22].

Several studies in animal models revealed that ethanol consumption during gestation increased *Cyp2e1* gene expression in the fetal liver [23] and activity in both the maternal and fetal liver, promoting oxidative stress in the feto–maternal unit [24]. Consistent with these results, the *Cyp2e1* maternal hepatic gene was overexpressed with ethanol consumption during gestation, and this effect was exacerbated by partial IGF-1 deficiency (Figure 6). However, no significant differences were found in the CYP2E1 protein expression levels in the maternal liver, probably due to a different regulation at the mRNA and protein levels of CYP2E1, as shown in previous studies [25,26]. Recent studies have disclosed that RNAs, e.g., mRNA and ribosomal RNA, are more susceptible to oxidative damage than DNA [27]. In this way, this different expression pattern in CYP2E1 mRNA and protein levels in the livers of pregnant dams could be a result of the oxidative environment promoted by ethanol consumption and IGF-1 deficiency, where a fast mRNA degradation is produced by ROS.

In the placenta, CYP2E1 is inducible by ethanol exposure and is localized in the syncytium of this organ, increasing its expression as the gestation progresses [28]. The results herein showed that CYP2E1 expression is decreased, especially in KO fetuses in the junctional zone, maybe because of placental alterations due to IGF-1 deficiency, suggesting that this deficiency is enough to generate changes in CYP2E1 expression, even with ethanol consumption.

4-HNE, a marker for lipid peroxidation, is increased along with CYP2E1 due to ethanol consumption, suppressing its generation with the IGF-1 exogenous treatment [29]. Here, no significant differences were found in 4-HNE protein expression in the liver of pregnant dams from all of the experimental groups. This suggests that more studies are needed to better determine how this lipid peroxidation marker, and hence the progress of an oxidative environment, alters intrauterine development, and if it is related to either partial IGF-1 deficiency or ethanol consumption during gestation or both.

In humans, ethanol consumption during pregnancy was reported to promote lipid peroxidation in the fetal liver and the placenta and reduce CAT, SOD, and GPx activities in both mothers and newborns [30], leading to oxidative stress that can affect the fetus. However, in the maternal liver from rats, ethanol reduces GPx activity [31] and increases SOD activity, whereas the effect on CAT activity is dose-dependent [32], all of this promotes the oxidative modification of liver proteins that could contribute to the adverse effects of ethanol observed in this tissue. Accordingly, in the present study, it was observed a significant increase in CAT activity with a nonsignificant decrease in both GPx and SOD activities in the livers of WT pregnant dams exposed to ethanol during pregnancy.

On the other hand, the IGF-1 effect on the expression and/or activities of antioxidant enzymes is notable. In studies using Ames dwarf mice, which present low levels of IGF-1 in serum, a diminished protein concentration in both SOD and GPx at the vascular level was observed, with induction on their expression after treatment with IGF-1 [33] but did not show changes in CAT expression, whereas some reports showed that the external administration of IGF-1 reduces the activities (CAT and GPx) and expression (SOD) of these enzymes in the livers of mice [34]. In this sense, herein was observed that IGF-1 deficient mice presented a significant increase in liver GPx activity, but no changes were observed in both CAT and SOD activities in the liver; more studies are needed to determine the effect of IGF-1 deficiency on these antioxidant enzymes in the liver, in order to clarify that whether the partial IGF-1 deficiency is promoting a reductive environment resulting in more activity or production of antioxidant enzymes. Additionally, the combination of both IGF-1 deficiency and ethanol consumption did not show any significant outcome on the antioxidant enzyme activities in the liver. Nevertheless, with regard to GPx, the same tendency (lower activity) was observed with ethanol exposure either in WT or IGF-1 deficient mice. These results indicate that the animal model used in the present study may not be the most appropriate for evaluating the effects of alcohol consumption during pregnancy.

CAT, SOD, and GPx, the major antioxidant enzymes capable of reducing ROS that causes oxidative stress, are present in the placenta. Studies in human and murine placentas show that ethanol consumption increases CAT and SOD activities, while GPx activity remains the same or decreases during pregnancy [35], suggesting that there is a repression of lipid peroxidation, thus protecting the fetus against ROS toxicity. The results herein hint, for the first time, that the chronic ethanol exposure of partially IGF-1-deficient mice during gestation increases CAT and GPx activities in the placentas. Of note, GPx placental activities are related to the extent of IGF-1 deficiency, highlighting the crucial role of this organ in the protection of the fetus against the oxidative stress generated both by pregnancy and external insults (Figure 6). Contrary, no differences were found in SOD activities in placentas from all groups, in accordance with what was found in the literature [36].

Actually, partial IGF-1 deficiency is a singular human condition where several physiological alterations occur due to the lack of this hormone. The most recognized partial IGF-1 deficiency conditions are cirrhosis, FGR, aging, and metabolic syndrome [37]. During development, the establishment of a proper intrauterine environment for fetal development is mandatory, a situation where IGF-1 plays a key role. To date, several case reports in women have shown that partial IGF-1 deficiency might be involved in alterations in the reproductive system and the attainment and maintenance of pregnancy [38,39,40], and also its implications in metabolism, which could alter the metabolism of xenobiotic substances such as ethanol. For this reason, the development of an animal model that resembles the physiological alterations due to partial IGF-1 deficiency is crucial for studying the mechanisms and molecular targets involved in its development, especially in critical periods such as pregnancy. In this sense, as seen before in reports from our research group, the mouse model used in the present study authentically represents the pathophysiological conditions associated with partial IGF1 deficiency, such as alterations of the hepatic architecture, mitochondrial damage, and lower body weight, among others, thus being a vital implement for the study of the pathological alterations associated with partial IGF-1 deficiency.

The present study has limitations. The results herein attempted to determine if partial IGF-1 deficiency exacerbated or ameliorated ethanol’s adverse effects throughout gestation. However, the observed effects were due mostly to partial IGF-1 deficiency during intrauterine development. The use of another animal model would allow us to better elucidate this scenario, and more studies are needed to determine placental alterations due to both IGF-1 deficiency and ethanol consumption. CYP2E1 regulation is complex, particularly in the present study, where two variables are impairing its expression, which is the reason why other biomarkers for ethanol consumption could be evaluated, e.g., ethyl glucuronide and ethyl sulfate. In addition, 4-HNE reported no significant differences between the groups, suggesting that other oxidative stress markers are needed, e.g., protein carbonyl content.

## 5. Conclusions

This study reveals that partial IGF-1 deficiency reduces body weight and CYP2E1 expression in the placentas of IGF-1 deficient mouse fetuses. Ethanol consumption during gestation diminishes body weight and food consumption during this period, increases placental weight and *Cyp2e1* expression in the livers of HZ pregnant dams, and blights the antioxidant system, suggesting impairments in placental development, which could lead to an altered nutrient exchange, resulting in malnutrition and poor fetal development. In this sense, a synergic effect between IGF-1 deficiency and ethanol consumption during gestation was observed in some fetal parameters, but more studies are needed to determine this asseveration.

## Figures and Tables

**Table 1:** Maternal body and liver weights on gestational day 19.

Experimental Group	Maternal Body Weight (G)	Maternal Liver Weight (G)	Ratio Maternal Liver/Body Weight	Food Consumption (G)/BODY Weight (G)
WT-Control (*n* = 6)	52.05 ± 1.21	2.73 ± 0.08	5.24%	0.175 ± 0.005
WT-Ethanol (*n* = 4)	51.73 ± 5.82	2.88 ± 0.10	5.57% *	0.140 ± 0.011 ^a,^*
HZ-Control (*n* = 6)	40.73 ± 1.28 ^b,c^	2.24 ± 0.07 ^c^	5.50%	0.188 ± 0.021 ^c^
HZ-Ethanol (*n* = 9)	42.76 ± 1.32 ^d^	2.49 ± 0.16	5.82% *	0.157 ± 0.004 ^d,^*

The statistical tests applied were Mann–Whitney U test or Student *t*-test to compare between WT and HZ mice and ethanol and water (control) treatments, and Kruskal–Wallis test or ANOVA to compare between all experimental groups. Differences were considered significant when *p < 0.05*. The letters showed the significant differences found: ^a^ *p < 0.01* WT-Ethanol vs. WT-Control; ^b^ *p < 0.05* HZ-Control vs. WT-Control; ^c^ *p < 0.05* HZ-Control vs. WT-Ethanol; ^d^ *p < 0.05* HZ-Ethanol vs. WT-Control; * *p < 0.05* Ethanol vs. Control.

**Table 2:** Weight and size measurements of pups and placentas at the end of the experimental protocol grouped by genotype and treatment.

Group	Offspring Genotype	Fetal Height (CM)	Fetal Weight (G)	Placental Weight (G)
Controls	WT (*n* = 9)	3.33 ± 0.066	1.08 ± 0.039	0.10 ± 0.007
	HZ (*n* = 32)	3.22 ± 0.035	1.03 ± 0.021	0.09 ± 0.004
	KO (*n* = 19)	2.75 ± 0.045 ^b,c^	0.72 ± 0.028 ^b,c^	0.08 ± 0.005
Ethanol exposure	WT (*n* = 23)	3.17 ± 0.041 ^a^	1.03 ± 0.024	0.10 ± 0.004
	HZ (*n* = 44)	3.18 ± 0.030	0.98 ± 0.018 ^a^	0.09 ± 0.003
	KO (*n* = 16)	2.74 ± 0.050 ^b,c^	0.66 ± 0.030 ^b,c^	0.10 ± 0.005 ^a^

The statistical tests used were Student *t*-test or U Mann–Whitney test to compare between two groups, ANOVA or Kruskal–Wallis test to compare between more than two groups, and general linear model to evaluate the effect of the two independent variables in the present study (ethanol consumption and IGF-1 deficiency). Differences were considered significant when *p* < 0.05. The letters showed the significant differences found: ^a^ *p* < 0.05 Ethanol vs. Control; ^b^ *p <* 0.001 KO vs. WT; ^c^ *p* < 0.001 KO vs. HZ.

## Data Availability

All of the raw data that support the findings of this study are available in Zenodo: https://doi.org/10.5281/zenodo.5932878.

## Supplementary Materials

The following supporting information can be downloaded at: https://www.mdpi.com/article/10.3390/biology11091264/s1, Table S1: Maternal parameters (maternal weight, average of food and beverage consumption) during the 14-days acclimation period to ethanol; Table S2: Maternal body weight at gestational day 1; Table S3: Densitometry readings obtained from each band in immunoblotting analysis for CYP2E1 and β-actin proteins in liver from pregnant dams; Table S4: Densitometry readings obtained from each band in immunoblotting analysis for 4-HNE and β-actin proteins in liver from pregnant dams; Figure S1: Immunoblot images for CYP2E1 and β-actin proteins in liver from pregnant dams; Figure S2: Immunoblot images for 4-HNE and β-actin proteins in liver from pregnant dams.
