# Supplementary material for: Effect of Ethanol Consumption on the Placenta and Liver of Partially IGF-1-Deficient Mice: The Role of Metabolism via CYP2E1 and the Antioxidant Enzyme System
Source: Biology (Basel). 2022 Aug 25;11(9):1264. doi: 10.3390/biology11091264 (PMC9495332; doi:10.3390/biology11091264)
Supplement: Supplementary file 1 [file biology-11-01264-s001.zip › biology-1852649-supplementary.pdf]

Supplementary material

Supplementary Table S1. Maternal parameters (maternal weight, average of food and beverage consumption) during the 14-days acclimation period to ethanol.

Experimental group	Weight gain (g)	Average food consumption (g)	Average beverage consumption (mL)
WT-Control (*n* = *6*)	-0.47 ± 0.39	4.61 ± 0.47	9.23 ± 0.96
WT-Ethanol (*n* = *4*)	-0.83 ± 1.24	5.46 ± 1.90	12.95 ± 0.99
HZ-Control (*n* = *6*)	-0.85 ± 0.40	4.36 ± 0.80	9.49 ± 1.05
HZ-Ethanol (*n* = *9*)	-0.94 ± 0.30	3.77 ± 0.34	12.09 ± 0.64

The statistical tests applied were Kruskal-Wallis test or ANOVA to compare between all experimental groups. Differences were considered significant at a level of $p < 0.05$: no significant differences were found.

Supplementary Table S2. Maternal body weight at gestational day 1.

Experimental group	Maternal body weight (g)
WT-Control (*n* = *6*)	28.10 ± 1.06
WT-Ethanol (*n* = *4*)	29.58 ± 1.55
HZ-Control (*n* = *6*)	$23.95 \pm 0.75^{a,b}$
HZ-Ethanol (*n* = *9*)	25.39 ± 0.48^{c}

The statistical test applied was ANOVA to compare between all experimental groups. Differences were considered significant at a level of $p < 0.05$. The letters showed the significant differences founded: $^{a}p < 0.05$ HZ-Control *vs* WT-Control; $^{b}p < 0.01$ HZ-Control *vs* WT-Ethanol; $^{c}p < 0.05$ HZ-Ethanol *vs* WT-Ethanol.

Supplementary Table S3. Densitometry readings obtained from each band in immunoblotting analysis for CYP2E1 and β-actin proteins in liver from pregnant dams.

Well in gel	Treatment	Mouse	Density CYP2E1	Density Actin	Actine normalized	Normalized experimental signal
1	WT+H2O	4544 HÍG	20664.075	15977.832	0.50	41474.13
2	WT+EtOH	4483 HÍG	29872.832	14994.933	0.47	63886.79
3	HZ+H2O	4447 HÍG	27043.125	18620.66	0.58	46573.73
4	HZ+EtOH	4428 HÍG	23872.468	11492.296	0.36	66614.64
5	WT+H2O	4422 HÍG	18486.125	18144.296	0.57	32672.69
6	WT+EtOH	4418 HÍG	28168.004	32068.539	1.00	28168.00
7	HZ+H2O	4446 HÍG	26660.489	25129.66	0.78	34022.07
8	HZ+EtOH	4403 HÍG	19801.024	24328.418	0.76	26100.75
1	WT+H2O	4502 HÍG	17562.803	28955.024	0.88	19911.77
2	WT+H2O	4402 HÍG	13610.953	31231.539	0.95	14306.56
3	HZ+H2O	4416 HÍG	16934.903	32827.66	1.00	16934.90
4	HZ+H2O	4430 HÍG	26686.56	28990.832	0.88	30218.43
5	HZ+EtOH	4424 HÍG	25438.832	25181.296	0.77	33163.40
6	HZ+EtOH	4437 HÍG	9329.125	23920.66	0.73	12802.88
1	HZ+H2O	4501 HÍG	30661.631	28245.317	0.56	54761.39
2	HZ+EtOH	4476 HÍG	29323.61	28850.024	0.57	51273.97
3	HZ+EtOH	4477 HÍG	31481.803	19405.681	0.38	81838.26
4	HZ+EtOH	4460 HÍG	22518.974	50445.877	1.00	22518.97
5	HZ+EtOH	4464 HÍG	27592.288	50445.877	1.00	27592.29

Supplementary Figure S1. Immunoblot images for CYP2E1 and β-actin proteins in liver from pregnant dams.

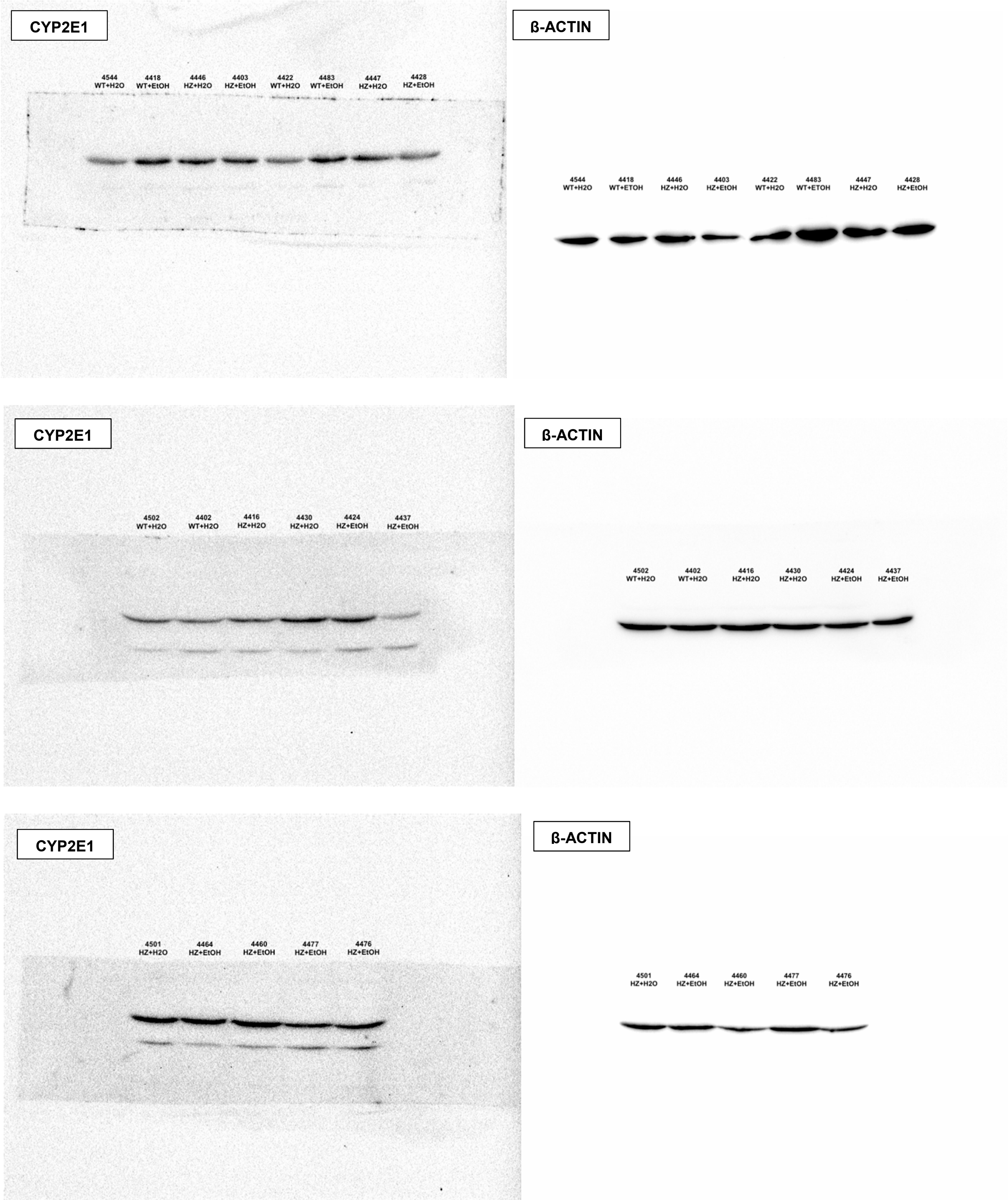

Supplementary Table S4. Densitometry readings obtained from each band in immunoblotting analysis for 4-HNE and β-actin proteins in liver from pregnant dams.

Well in gel	Treatment	Mouse	Density 4-HNE	Density Actin	Actine normalized	Normalized experimental signal
1	WT+H2O	4544 HÍG	8229.811	10104.276	0.35	23191.38
2	WT+EtOH	4483 HÍG	20078.439	12693.154	0.45	45040.41
3	HZ+H2O	4447 HÍG	17634.347	15327.447	0.54	32759.06
4	HZ+EtOH	4428 HÍG	15816.782	13193.497	0.46	34135.02
5	WT+H2O	4422 HÍG	19766.468	15694.205	0.55	35861.76
6	WT+EtOH	4418 HÍG	29704.61	27707.861	0.97	30525.50
7	HZ+H2O	4446 HÍG	20111.711	22428.74	0.79	25532.07
8	HZ+EtOH	4403 HÍG	10899.054	28473.569	1.00	10899.05
1	WT+H2O	4502 HÍG	8664.882	15695.225	0.65	13342.00
2	WT+H2O	4402 HÍG	23899.317	24167.175	1.00	23899.32
3	HZ+H2O	4416 HÍG	19244.489	23454.468	0.97	19829.27
4	HZ+H2O	4430 HÍG	5870.953	11769.225	0.49	12055.54
5	HZ+EtOH	4424 HÍG	4151.711	6900.861	0.29	14539.51
6	HZ+EtOH	4437 HÍG	7305.004	14868.518	0.62	11873.50
1	HZ+H2O	4501 HÍG	22177.388	19536.024	0.77	28801.86
2	HZ+EtOH	4476 HÍG	9158.731	11826.146	0.47	19648.91
3	HZ+EtOH	4477 HÍG	19366.095	13305.439	0.52	36928.29
4	HZ+EtOH	4460 HÍG	26484.803	25371.51	1.00	26484.80
5	HZ+EtOH	4464 HÍG	15308.024	11942.439	0.47	32521.64

Supplementary Figure S2. Immunoblot images for 4-HNE and β-actin proteins in liver from pregnant dams.

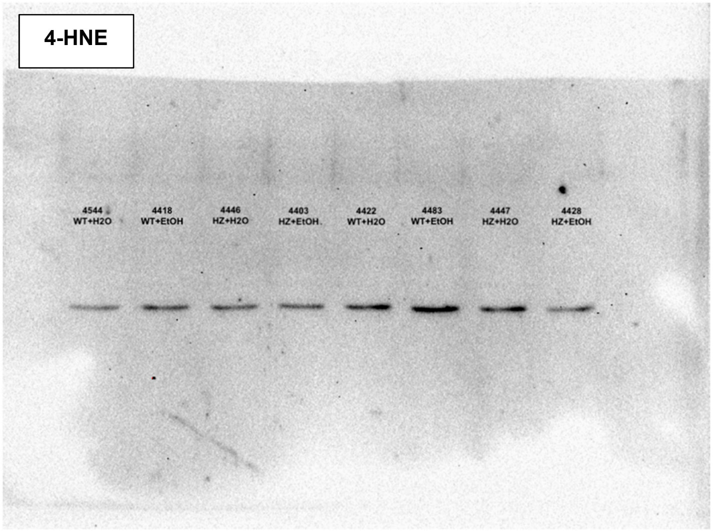

β-ACTIN

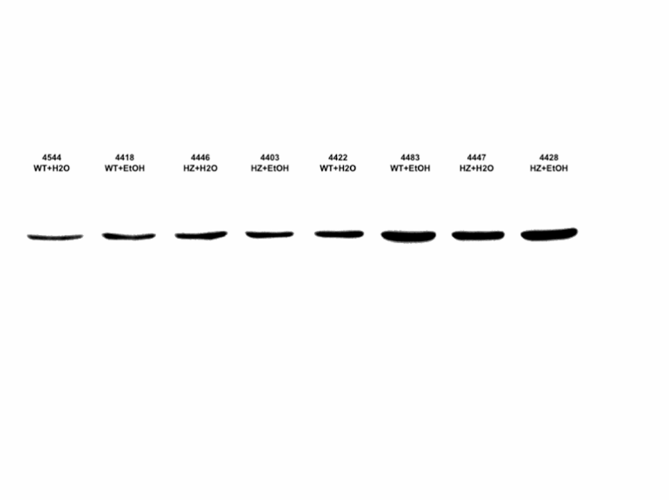

4-HNE

β-ACTIN

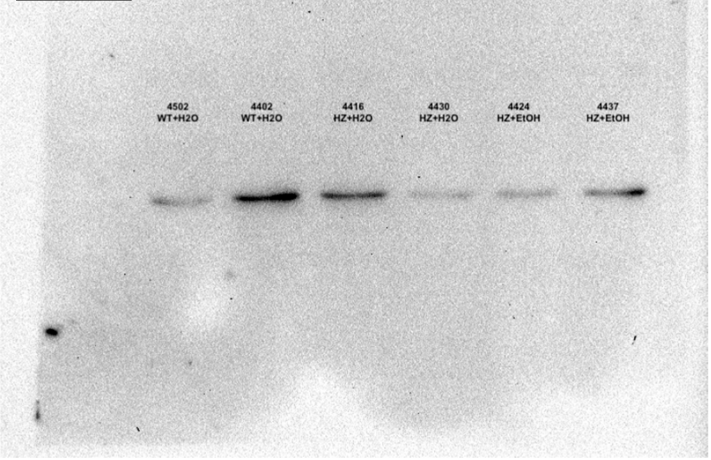
4502 WT+H2O
4402 WT+H2O
4416 HZ+H2O
4430 HZ+H2O
4424 HZ+EtOH
4437 HZ+EtOH

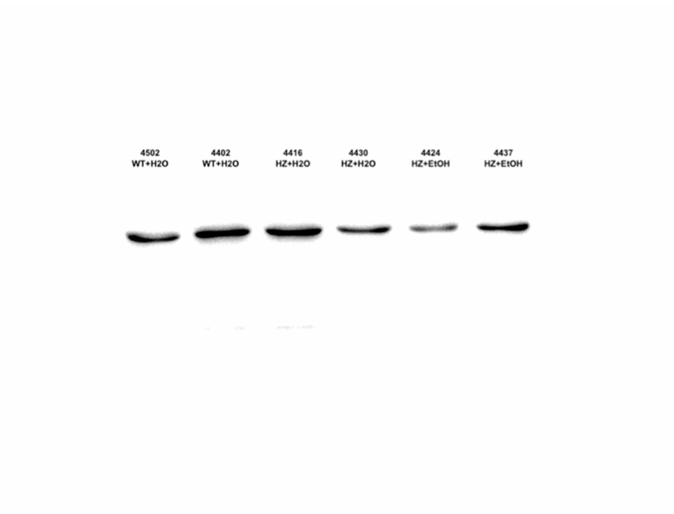
4502 WT+H2O
4402 WT+H2O
4416 HZ+H2O
4430 HZ+H2O
4424 HZ+EtOH
4437 HZ+EtOH

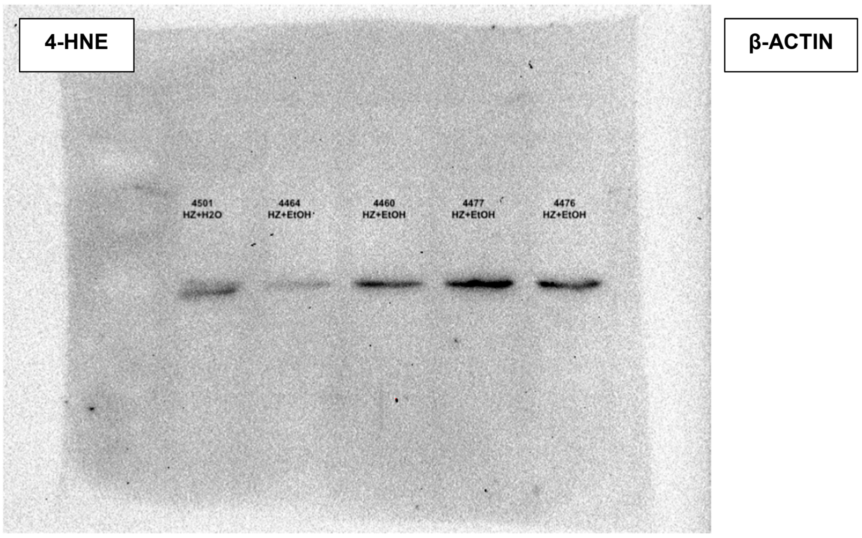
4-HNE
4501 HZ+H2O
4464 HZ+EtOH
4460 HZ+EtOH
4477 HZ+EtOH
4476 HZ+EtOH

β-ACTIN

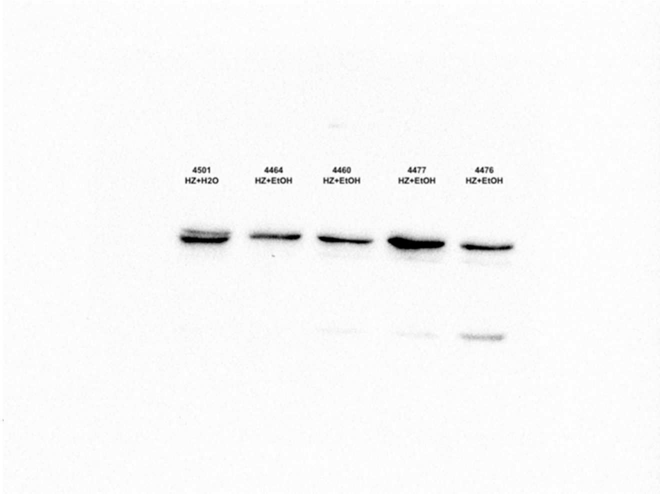
4501 HZ+H2O
4464 HZ+EtOH
4460 HZ+EtOH
4477 HZ+EtOH
4476 HZ+EtOH
